# Soft and flexible piezoelectric smart patch for vascular graft monitoring based on Aluminum Nitride thin film

**DOI:** 10.1038/s41598-019-44784-1

**Published:** 2019-06-10

**Authors:** L. Natta, V. M. Mastronardi, F. Guido, L. Algieri, S. Puce, F. Pisano, F. Rizzi, R. Pulli, A. Qualtieri, M. De Vittorio

**Affiliations:** 1 Center for Biomolecular Nanotechnologies, Istituto Italiano di Tecnologia, 73010 Arnesano, Le Italy; 2 Università del Salento, 73100 Lecce, Italy; 3 Università di Bari ‘Aldo Moro’, Department of vascular surgery, 70121 Bari, Italy

**Keywords:** Disease prevention, Biomedical engineering

## Abstract

Vascular grafts are artificial conduits properly designed to substitute a diseased blood vessel. However prosthetic fail can occur without premonitory symptoms. Continuous monitoring of the system can provide useful information not only to extend the graft’s life but also to optimize the patient’s therapy. In this respect, various techniques have been used, but all of them affect the mechanical properties of the artificial vessel. To overcome these drawbacks, an ultrathin and flexible smart patch based on piezoelectric Aluminum Nitride (AlN) integrated on the extraluminal surface of the prosthesis is presented. The sensor can be conformally wrapped around the external surface of the prosthesis. Its design, mechanical properties and dimensions are properly characterized and optimized in order to maximize performances and to avoid any interference with the graft structure during its activity. The sensorized graft is tested *in vitro* using a pulsatile recirculating flow system that mimics the physiological and pathological blood flow conditions. In this way, the ability of the device to measure real-time variations of the hemodynamics parameters has been tested. The obtained high sensitivity of 0.012 V Pa^−1^ m^−2^, joint to the inherent biocompatibility and non-toxicity of the used materials, demonstrates that the device can successfully monitor the prosthesis functioning under different conditions, opening new perspectives for real-time vascular graft surveillance.

## Introduction

A prosthetic vascular graft is an artificial conduit, designed on the patient’s arterial anatomy, to bypass a diseased vessel. Although recent technological advances have allowed improvements of prosthetic graft performances, failures can occur without any premonitory symptoms^1^. In fact, despite the “failing-graft” has a hemodynamically compromised blood flow, no evident clinical signs are observed, and its continuous asymptomatic deterioration can result in thrombosis and possible ischemia of the treated area. Of note, the treatment of a thrombosed graft is a really complex process, which can lead to an increased postoperative morbidity and, in the worst case scenario, to patient mortality. A bypass surgery is mandatory when the graft is completely occluded, whereas an easier endovascular procedure for a local angioplasty is sufficient to restore the physiological blood flow in case of only partial occlusion^2^. However, even if most surgical practices have incorporated a follow up program of graft surveillance to increase the “failing-graft” recognition^3^, no single blood flow sensing technique has been adopted, in clinical practice, for real time monitoring of the prosthetic graft^4^. The follow-up surveillance after the implantation is currently based on ultrasound protocols, computed tomography scan and angiograms at defined time intervals in order to evaluate parameters such as graft flow velocity, variation in lumen dimensions and blood flow pressure^4,5^. However, the prevention of failures is not exceptionally efficient nowadays^4,6,7^. In this respect, the current techniques come with procedural morbidity, possible exposure to nephrotoxic contrast agent and are time consuming and expensive. All together, these factors limit their wide application and the potential benefit for early diagnosis^4^. To resolve these issues, a continuous monitoring of the vascular graft is extremely important to extend its lifetime, as well as to provide vital information for early failure warning. In this respect new solutions based on different analysis techniques have been investigated to monitor the graft status^2,4,8,9^. One approach involves the use a Micro-Electro-Mechanic System (MEMS) based pressure sensor, inserted in direct contact with the blood-stream, which causes the deformation of a strain gauge^4,9^. Although these pressure sensors can detect the hemodynamic variation with a good sensitivity, they require a voltage supply and traditional electronic components which alters the structure of the graft itself. Furthermore, the direct contact of the sensor with the blood can induce localized turbulences in the blood flow and can generate response to the “foreign body”. More recently, a new class of flexible sensors has been developed to be directly mounted on the external graft surface, so to follow the vascular graft expansion due to the pulsatile blood flow. To do this, a possible approach uses a flexible strain sensor composed of structured polydimethylsiloxane (PDMS) with an embedded conductive PDMS to measure graft wall deflection^8^. Yet, the dimensions of this sensor still affect the mechanical properties of the graft. An innovative approach is based on the piezoelectric effect. Indeed, the ability of piezoelectric based sensors to instantaneously generate an output voltage as an effect of response to rapid variations of dynamic pressure makes them very appealing for monitoring the blood flow. Different types of piezoelectric sensors have been used to evaluate different hemodynamic parameters for both on skin and implantable applications^2,10,11^. In particular, a sensor unit based on two polyvinylidene difluoride (PVDF) sensors (28 μm) encapsulated in Mylar® foil has been developed^2^. The system wrapped around the graft evaluates flow variations in both *in vitro* and *in vivo* models. The reported results show how the piezoelectric transduction is really useful to monitor hemodynamic variations and the presence of small occlusions in the graft, but the sensor dimensions with its high thickness still affects the mechanical behavior of the graft structure.

Here we demonstrate an ultrathin, lightweight and flexible smart microsystem, based on piezoelectric Aluminum Nitride (AlN), integrated on the extraluminal surface of prosthesis, for the continuous monitoring of the vascular graft. The piezoelectric device, wrapped on the extraluminal surface of the graft, generates an electric response due to the wall deformation, providing important information about the blood flow hemodynamics in the artificial vessel. The proposed sensor is developed as a smart patch composed by the sensitive piezoelectric area, embedded between two electrodes and inserted in an ultrathin, flexible polymeric matrix made of polyimide (PI) and a thin layer of PDMS. Importantly, the AlN is inherently biocompatible and non-toxic^12^, with high electromechanical coupling^13^ and intrinsic high resistivity^14^, making it one of the primary choices for the development of an implantable piezoelectric sensor. The whole fabrication process trades on traditional microfabrication techniques, allowing a simple engineering of the geometry and definition of the mechanical properties of the final device, to improve the matching with the structure of the artificial vessel, without affecting its behavior.

This work encompasses the fabrication and characterization of the flexible smart patch, the finite element modeling (FEM) and shows the experimental results derived from the study of the graft-sensor system, exploiting an *ad-hoc* measurement set-up.

## Results and Discussion

The flexible piezoelectric smart patch consists of a sensing element with a rectangular shape whose active area is composed of a multilayered stack consisting of aluminum nitride-interlayer (AlN-IL), molybdenum bottom electrode (Mo), piezoelectric aluminum nitride (AlN) and molybdenum top electrode (Mo). A dynamic external stress, applied to the sensing element by the cyclic expansion and contraction of the graft walls, allows the smart patch to generate a proportional electrical peak voltage by virtue of the direct piezoelectric effect. This voltage output (V_piezo_) is then acquired to extract information about the status of the graft. In this work, the quality of the piezoelectric AlN deposited on polyimide was verified by means of morphological analysis and piezo force microscopy. The representative AlN flexible sensor – named PSP-20 (piezoelectric smart patch-20 mm^2^) –, having a piezoelectric area of about 4 × 5 mm^2^, was mechanically and electrically tested. Finally, the sensor was used to continuously monitor the status of a prosthetic graft.

**Figure 1:**
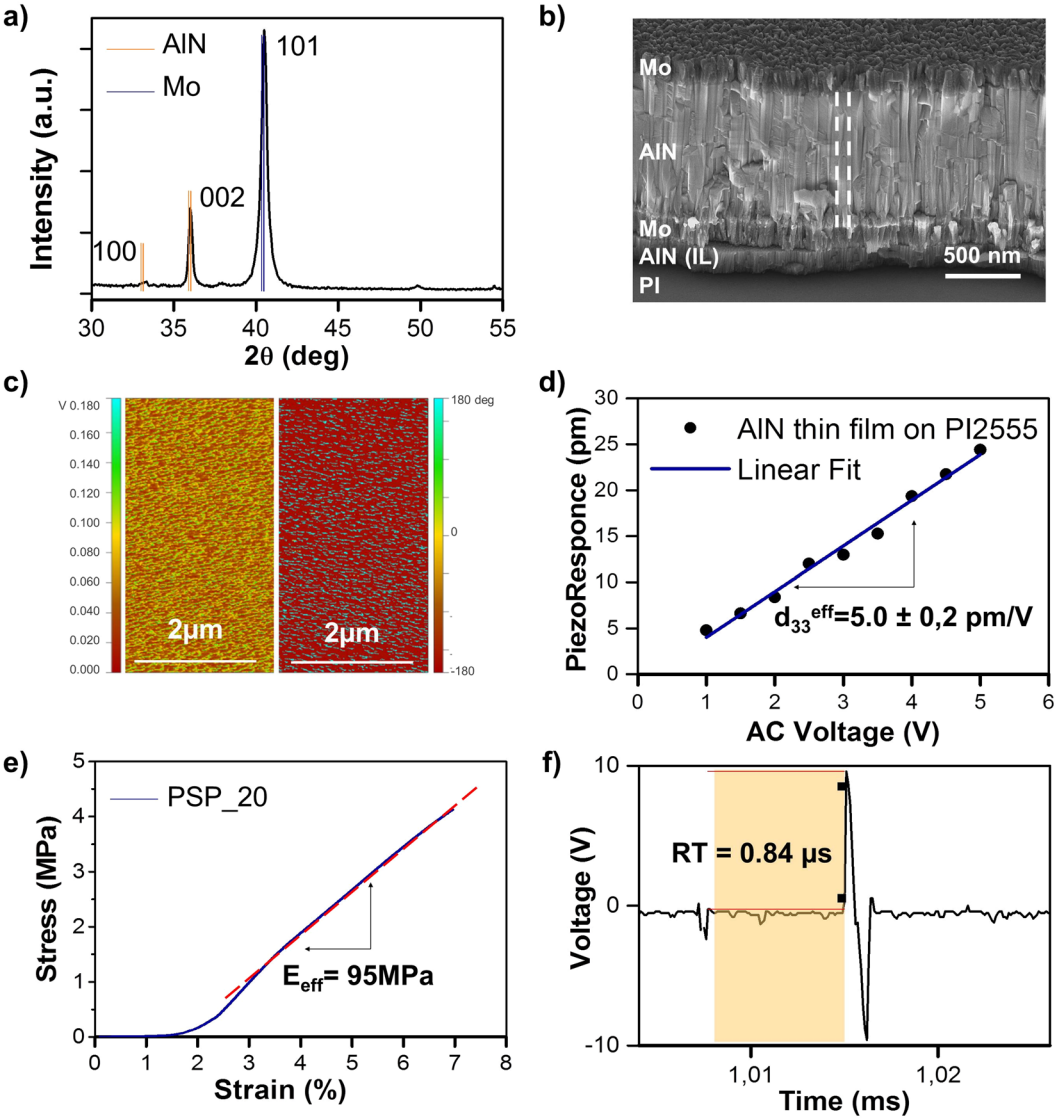
SPS-20 characterization. (**a**) XRD spectrum of the AlN thin film deposited on PI substrate, (**b**) SEM image showing the cross-section of the PSP-20, with the definition of the stack layers. The dashed line shows the direction of the columnar growth of the grain, (**c**) Vertical PFM amplitude (left) and phase (right), (**d**) Average piezoresponce values versus the applied voltage. The linear fit of the experimental values is reported for the d_33_ evaluation (**e**) experimental Stress-Strain diagram is reported. The effective Young modulus value of the entire heterostructure is measured as the slope of the linear region (red dashed line), (**f**) PSP-20 response time to the application of an instantaneous normal force of 50 kPa.

The morphological structure and crystalline orientation of the AlN thin film deposited on polyimide substrate after the transfer process from the rigid substrate, were evaluated with particular attention to the grain orientation along the *c*-axis growth direction. For this purpose, X-Rays diffractometry (XRD, D8-Discover Bruker diffractometer) and scanning electron microscopy (SEM, NanoLab 600i SEM/FIB, FEI) were used. Figure 1a shows the curve of diffracted intensities, collected in the *θ*–*2θ* scan mode, in which a sharp peak at 36° is observed, corresponding to (002) crystal orientation of the AlN hexagonal structure. A second, lower peak is also evident at about 33°corresponding to the (100) crystal orientation. The intensity ratio between these two peaks confirms the preferential growth direction along the *c*-axis, suggesting a well oriented AlN, grown by sputtering on flexible polyimide substrate and good piezoelectric properties of the thin film.

Visual inspection with SEM shows a good continuity through the different grains, as well as a clean separation between the layers (Fig. 1b).

Piezo force microscopy (PFM, CSI Nano-Observer AFM microscope) was used to evaluate the piezoelectric response of the deposited AlN. This technique exploits the application of a modulated AC voltage between the bottom Mo electrode of the tested sample and the conductive tip of the instrument, to measure the elastic deformation of thin film. Figure 1c shows the piezoresponse amplitude and phase images of the sputtered AlN thin film, biased at a constant voltage of 5 V. These two images confirmed a uniform distribution of the piezoelectric coefficient magnitude through the different grains, mantaining the same polarization.

From the piezoresponse amplitude curve (Fig. 1d) - carried out at different values of AC, from 0 V up to 5 V - the effective piezoelectric coefficient *d*_33_^*eff*^ is measured as the slope of the piezoresponse amplitude versus applied voltage. The measured value of 5.0 ± 0.2 pm/V is higher than that reported for AlN sputtered on polymeric substrates^15,16^.

PSP-20 was then characterized in order to evaluate its mechanical properties. Flexibility is in fact a key parameter and an elastic modulus lower than the modulus of the prosthetic graft guarantees sensitivity to small deformations of the graft surface without affecting, at the same time, the normal behavior of the graft itself. In this respect, the effective Young’s modulus of the whole heterostructure was measured using a dynamic mechanical analyzer (DMA, TA Q800), with an uniaxial tensile testing. In this test, the sensor has been subjected to a controlled tension, with a force range varied from 0 up to 1 N, with a ramp of 0.1 N/min. The considered sample has a rectangular shape with a thickness of 17 µm, a width of 9.57 mm and a measured length of about 17 mm. The Young’s modulus, extrapolated from the slope of the linear zone of the stress-strain curve (dashed line in Fig. 1e), was about 95 MPa, which is an order of magnitude lower than the modulus of the graft (E_graft_ = 0.5 GPa), as desired^17^. Finally, using the DMA compression clamp tool, a periodic and impulsive normal force was applied in order to measure the response time of the PSP-20. A constant pressure of 50 kPa was applied by the use of an hemispherical tip whereas the sensor was connected to the oscilloscope (Tektronix, MDO4000). The corresponding generated voltage acquired showed that the sensor exhibited a very fast response to the impulsive applied force, whose value, being 0.84 µs, has been evaluated as the rise time between the 10% and 90% of the ascending edge (Fig. 1f).

Subsequently, a FEM model (built by COMSOL Multiphysics software) has been developed to verify the responsivity of the sensor to a pressure variation. The simulations have been set up combining structural mechanics with electrodynamics, describing the material piezoelectricity through a strain-charge matrix. The structure was defined, following the real sensor structure, by a superposition of the AlN-IL/Mo/AlN/Mo stack, embedded between two polyimide layers on a PDMS structural layer, with a thickness of about 20 μm. The whole patch is, then subsequnetly, attached to a PDMS deformable membrane (thickness 500 μm), used as substrate mimicking the soft system (skin, vessel, graft). Between the PDMS membrane and the smart piezoelectric patch, an adhesion layer of Poly(vinyl alcohol) (PVA) has been inserted in the simulations, in order to account for the glue to which the smart patch is attached. Nonlinearity in PDMS behavior has been introduced in FEM simulations. To better validate the simulations, the effective Young’s modulus of the stack (E_eff_ ≈ 95 MPa) - previously measured by the DMA tensile testing - has been introduced in the FEM description in each PDMS/PI/AlN-IL/Mo/AlN/Mo stack layer materials data. Similarly, the PDMS membrane material data with a Young’s modulus of E_PDMS_ ≈ 2 MPa, measured by DMA, was used. PVA material data were acquired from literature^18^ while its layer thickness was measured by profilometer (55 μm). Because of its wurzite structure, AIN has been assumed to be in-plane isotropic and the compliance matrix has been formulated accordingly. Consequently, the effective piezoelectric coefficient d_33_^eff^ of the AlN layers, measured by PFM, has been inserted in the Strain-Charge matrix. Table 1 shows materials’ parameters and layer thicknesses used in the simulation.

**Table 1:** List of the materials data inserted in the FEM simulation.

Material	Density (Kg/m^3^)	Poisson ratio	Young Modulus (MPa)	Thickness (µm)
PDMS membrane	970	0.49	2	500
PVA	1.26	0.44	3000	55
PDMS_stack	970	0.49	95 (effective)	15
Polyimide	1300	0.49	95 (effective)	4
Mo_stack	10200	0.31	95 (effective)	0.2
AlN_stack	3300	—	95 (effective)	1
AlN_Interlayer	3300	—	95 (effective)	0.1

Density value, poisson ratio, Young modulus, and thickness used in the implemeted model for the FEM simulation.

**Figure 2:**
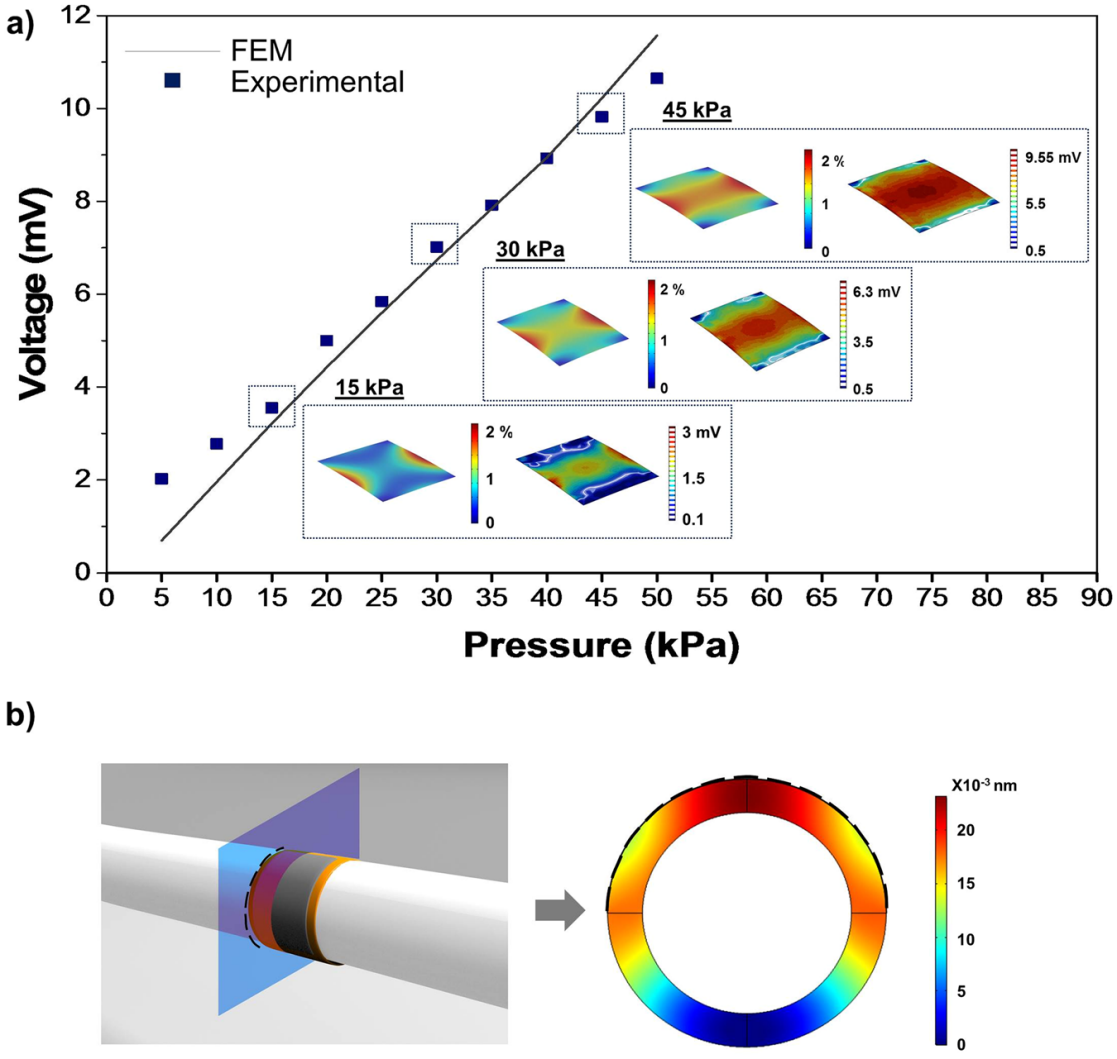
FEM modeling and piezoelectric sensor sensitivity. (**a**) Sensor response to a pressure variation between 5 kPa and 50 kPa. The Inset images show the strain distribution along the Z axes of the AlN layer at 15, 30, 45 kPa (the reported values has been chosen as representative of the entire pressure range) correlated to the electrical response map. (**b**) Displacement distribution on the transverse cross-section of the sensorized vascular graft due to the application of the PSP-20 on the extraluminal surface (black dotted line).

According to the literature^19^, the pressure produced by the human body movement is distributed between 1 to 100 kPa and, in particular, the pressure range for blood vessels pulses is between 5 to 50 kPa. FEM simulations were performed in this range, by applying a parametric sweep with a step of 5 kPa and, for each pressure value. A stationary study has also been carried out to estimate the electrical response of the piezoelectric layer. The computational results demonstrated that the resulting strain on the AlN thin film is directly correlated with the applied pressure on the membrane and that the spatial distribution of the strain depends on the geometrical characteristics of the patch. Due to the direct piezoelectric effect, the progressive increasing of the mechanical strain (strictly related to the increase of pressure in the vessel) corresponds to an increase of the electrical response in terms of output voltage generated by the AlN, as reported in Fig. 2a (inset). This correlation can be written as^20^:

1 $${V}_{piezo}=\alpha {A}_{contact}P$$

where P is the applied pressure, A_contact_ is the contact area between the smart patch and the PDMS pushing membrane and α is a parameter that depends on the materials’ constant and on the stack’s deformation mechanics. These conditions have been replicated in the laboratory by applying the same pressure range on the PDMS supporting membrane, deforming the piezoelectric sensor and directly measuring the generated open circuit voltage with an oscilloscope. The obtained results, reported in Fig. 2a, showed that the piezoelectric thin film generates a voltage that linearly increases with the applied pressure on the PDMS supporting membrane. The experimental results are in good agreement with the FEM estimations and strongly support the effective description of the patch stack, with a single effective Young’s modulus and the insertion of an adhesion layer in the simulated system. The output voltage generated by the PSP-20, without any amplification or filters, consistently increases following a linear trend in the entire pressure range, with an α coefficient of 0.012 V Pa^−1^ m^−2^.

A model of the sensorized vascular graft has been developed in order to simulate the deformation induced on the graft due to the attachment of the sensor on its extraluminal surface. Following the real structure of the system, the geometry has been defined building the PSP-20 on the surface of the prosthesis. The graft has been modeled as a polytetrafluoroethylene (PTFE) ring with a thickness of 1 mm. To provide the model with realistic features, the effective mechanical properties of the stack (reported in Table 1) were used in the FEM description of all the smart-patch layer materials’ data. PTFE materials data were retrieved from the literature^21^. The simulations have been set up exploiting structural mechanics describing the mechanical behavior of the patch using the “plate theory”^22^. In this respect the bending rigidity of the PSP-20 was described by the Eq. (2):

2 $$D=\frac{2E{h}^{3}}{3(1-{\nu }^{2})}$$

where E is the Young’s modulus, h the patch thickness and ν is the Poisson’s ratio. It is demonstrated that the 20 μm-thick implant, does not affect the whole structure of the vascular graft. Indeed, a total difference in the radial displacement of 0.02 nm has been observed when the patch is attached to the graft, as reported in Fig. 2b where the patch is highlighted with a black dashed line.

To verify the ability of the sensor to sense the variation of hemodynamics conditions in the graft and to evaluate the presence of different percentages of occlusion, a dedicated measurement set up has been developed. The sensor has been wrapped in the external surface of a commercial PTFE graft (that substitutes the PDMS supporting membrane used in the previuos tests) in order to perform a comprehensive characterization of the device response as a function of the graft behavior.

**Figure 3:**
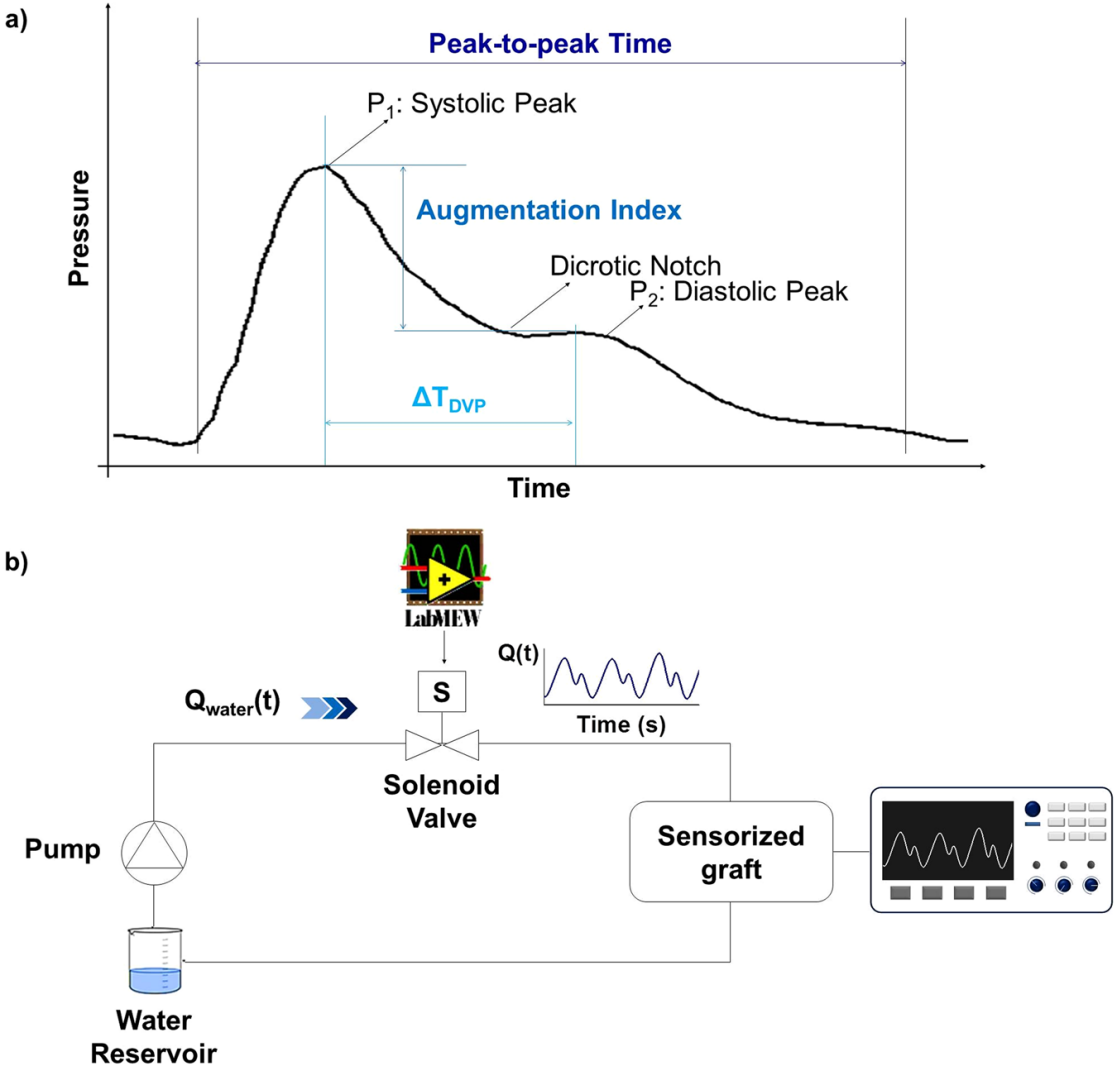
Pulse waveform signal and measurement set up. (**a**) A typical shape of the pulse wave signal and its characteristic parameters, defined as systolic peak P_1_, diastolic peak P_2_ and dicrotic notch. Augmentation index, ΔT_DVP_, and peak-to-peak features are reported, (**b**) schematic illustration of the measurement set-up for the on graft measurements used for the *in-vitro* experiments.

Generally, the most common way to evaluate the hemodynamics in a vessel is to study the pulse wave signal (Fig. 3a) recorded at different locations (the most common from the arm, recording brachial pressure by the sphygmomanometer)^23^. As a matter of fact, this signal is useful for recording information about the blood pressure and the analysis of its waveform provides important clinical information. The pulse wave signal is commonly divided in two main parts defined as anacrotic phase (the rising edge of the pulse) and catacrotic phase (the falling edge of the pulse) which corresponds to different moments of the cardiac cycle^24^. The corresponding curve is characterized by the presence of two peaks, related to systole (SP) and diastole (DP), respectively, and a dicrotic notch (DN) between them. A distortion in the wave profile and, in particular, a variation of amplitude and position of these parameters are significant signs of an undesired variation in the patient hemodynamics, such as atherosclerosis or different heart dysfunctions^23^. In this respect, recording specific alterations of these signals on vascular grafts allows retrieving information on the graft status and highly predictive signs of prosthetic failures. According to these considerations, an *ad hoc* measurement set up has been developed, in order to pump a flow into a sensorized graft and mimic the behavior of a real arterial blood flow. In particular, the continuous sequence of systolic and diastolic peaks with the corresponding dicrotic notch in between have been successfully simulated. Moreover, their specific amplitude and generation time have been varied properly. The setup, presented in Fig. 3b, involved the sensorized graft inserted in a recirculating pulsatile system where water was pushed in by a pump through a solenoid valve whose flow was modulated by changing the valve opening. The system was set to a flow of about 350 ml/min and a tube with flow resistance of about 40 mmHg has been used in order to be consistent with the reported values of blood flow that typically moves inside an artery^25^. The solenoid valve was controlled by a power supply driven by a LabVIEW© script. In this way, a cyclic routine was achieved that controlled the water flow by properly varying the opening percentage and relative time interval of the valve channel, inducing the creation of previously mentioned parameters (both systolic and diastolic peaks and dicrotic notch). The output response of the sensor as a voltage signal has been acquired and recorded with an oscilloscope.

The PSP-20 output has been acquired in different hemodynamics conditions, properly replicated by the measurement set up by controlling the following parameters:

(i) the variation of the peak-to-peak interval, closely related to the R-R interval of the electrocardiogram (ECG) and useful to define the duration of a complete heart cycle and the corresponding flow rate. Moreover, a continuous control of this parameter is fundamental because its variation can influence the usefulness of some indexes (to be discussed later)^26^.
(ii) the variation of the Augmentation Index (AI); this parameter provides information on the stiffness of a vessel – indeed, arterial stiffness is an important marker of cardiovascular risk and atherosclerosis^5,26^ – allowing the recognition of modifications in the graft wall compliance due to the formation of a plaque inside the vessel. This index is defined as the ratio between the diastolic and systolic peaks, respectively defined as P_2_ and P_1_, (Eq. (3)) of the pulse wave signal^24^.
  3 $$AI( \% )={P}_{2}/{P}_{1}\ast 100$$
(iii) the variation of the time interval between the two peaks (ΔT_DVP_). This index is related to the transit time of pressure wave, from the central cardiovascular system to the periphery and its reflection. It is strictly correlated to AI and provides additional information about the mechanical properties of the vessel and the blood pressure (Eq. (4)) ^20,24^,
  4 $${\rm{\Delta }}{T}_{DVP}={t}_{{P}_{2}}-{t}_{{P}_{1}}$$
(iv) the presence of different percentages of occlusion in the vessel, since restenosis appears to be the main cause of vascular graft failing after one year^27^.

**Figure 4:**
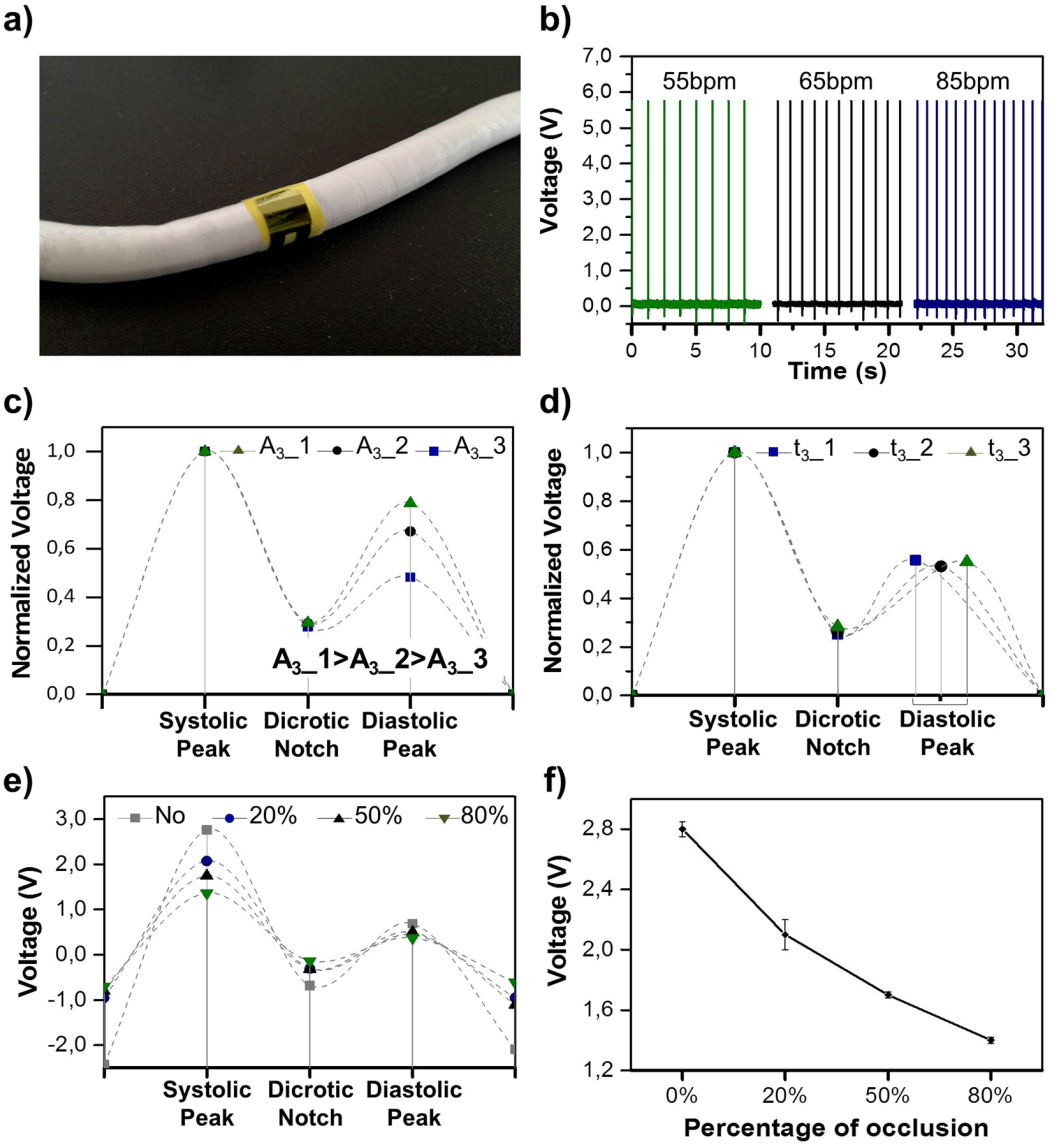
*In-vitro* on graft measurements. (**a**) Image of the e-PTFE graft integrated with the flexible smart patch (PSP-20) sensor for the hemodynamics parameter monitoring. (**b**) Generated output voltage of the PSP-20 sensor under different flow rate conditions, (**c**) voltage response of the sensor due to a Augmentation Index variation obtained changing the aperture percentage of the solenoid valve at the diastolic peak. The dashed line report an ideal fit of the pulse wave signal, (**d**) voltage response of the sensor due to a variation of the time interval between the systolic and diastolic peak, obtained changing the aperture time of the solenoid valve at the diastolic peak. The dashed line report an ideal fit of the pulse wave signal, (**e**) voltage response of the sensor due to different percentage of occlusion into the vascular graft. The dashed line report an ideal fit of the pulse wave signal, (**f**) voltage response vs percentage of occulsion into the graft prostethis.

Flow signals are reproducibly obtained from the sensor on the graft as a function of flow parameters. Firstly, the PSP-20 device attached to the graft (Fig. 4a) generates a signal that is closely correlated to the flow variation inside the graft, controlled by varying the peak-to-peak time interval. In particular, the pulse frequency of the flow has been varied in an interval of physiological values, between 55 and 85 bpm. As reported in Fig. 4b, the sensor is able to detect the frequency variation of the flow, displaying no noticeable fluctuation in the signal amplitude: this is an indication of the stability of the generated signal.

Despite the sensor measured response time (0.84 µs), the whole system (PSP-20 and graft) is able to detect frequency variations with an overall response time of about 250 μs, due to the damping effects induced by the graft deformation. This response time respects the common requirements for flow rate monitoring and it is comparable with the recently reported results about flexible piezoelectric sensors for blood flow analysis^10,11^.

In order to simulate the variation of the augmentation index, an induced change of the P_2_/P_1_ ratio has been imposed inside the graft. For this purpose, the opening percentage of the solenoid valve has been varied accordingly to the peak amplitude variation of the pulse wave shape. In particular, a variation of P_2_ amplitude has been enforced. The variation of this parameter is more indicative in terms of clinical evaluation. Indeed, reduced compliance causes an earlier return of the reflected wave, and this phenomenon is commonly translated in a modification of the P_2_ amplitude. Considering that, when the elastic arteries become stiffer there is a consequent increase of the augmentation index value^28^ – which is expected for the artificial vessel that mimics the arterial properties and behavior - the P_2_ amplitude has been varied in order to modify the AI, starting from a value of about 0.45, compatible with a safe vessel, until 0.80, typical of a highly stiff one. The plot reported in Fig. 4c shows that the voltage generated by the PSP-20, in correspondence with the diastolic peak, proportionally increases with the increase of the solenoid valve opening percentage and the consequent increase of the pressure applied to the graft wall by the water flow. This results establishes that the sensor, detecting the variation in the AI, can effectively acquire information regarding arteriosclerosis and heart diseases.

A similar approach has been used to evaluate the feasibility of the PSP-20 for the measurement of the transit time of pressure wave, namely ΔT_DVP_. A variation of the time interval between the P_1_ and P_2_ peaks has been imposed by modifying the opening time of the outlet channel of the solenoid valve. From the analysis of the shifted peaks, it is shown that the sensor can detect even small variations of the time interval between P_1_ and P_2_, without any undesired variation in the output voltage amplitude of the sensor, in good agreement with the imposed time step of the solenoid valve of about 50 ms (Fig. 4d). These results, together with the analysis of the augmentation index AI, demonstrate the ability of the PSP-20 to detect pulse wave shape modifications that are typically correlated to the presence of plaques in the inner walls of the graft.

Finally, the presence and the quantification of different percentages of occlusion in the vessel have been additionally evaluated by the PSP-20 attached to the graft. Different levels of stenosis have been introduced in the flow system by the use of a surgical forceps that can opportunely obstruct the graft, in a controlled way, between the 20% and the 80% of the initial diameter. In particular, as shown in Fig. 4e, the generated voltage varies congruently with the reduction of the vessel diameter, showing a flattening of the trend line with the increase of the occlusion percentages. This result demonstrates that the sensor is able to recognize the flow patterns in the vessel related to the presence of the stenosis, as created in the model, properly differentiating the different degrees of induced occlusion, as is shown in Fig. 4f. In this way, even a small occlusion can be successfully detected.

## Conclusion

The results presented here demonstrate the possibility to exploit an ultrathin and flexible piezoelectric sensor to continuously monitor the status of a vascular graft during its normal operation and under different hemodynamics conditions. When coupled with the external surface of the prosthesis, the sensor offers an accurate measurement of the motion of the graft wall due to the blood pulse wave in the vessel. In this way, it provides important information about the pathological variation of this signal related to the presence of a stiffening of the vessel due to a post-surgical restenosis, and even about the percentage of this occlusion. The generated data can alert the patient and, as a consequence, the physician of a change in graft function, allowing for an early detection of the problem and for the definition of a proper intervention before the complete graft failure. The thin and flexible structure of the device, together with its really sensitive and predictable response, offers high performances without affecting the structure of the vascular graft and modifying its behavior. Furthermore, the intrinsic biocompatibility and non-toxicity of the AlN and of all the different material used in the fabrication process, makes the system well suited to be implanted in the human body. In this respect, we are currently investigating the possibility to join a wireless flexible transmission system sending the signal from the inner to the outer part of the body, opening new perspective for real time vascular graft surveillance and even for monitoring of diseased blood vessels.

## Methods

**Figure 5:**
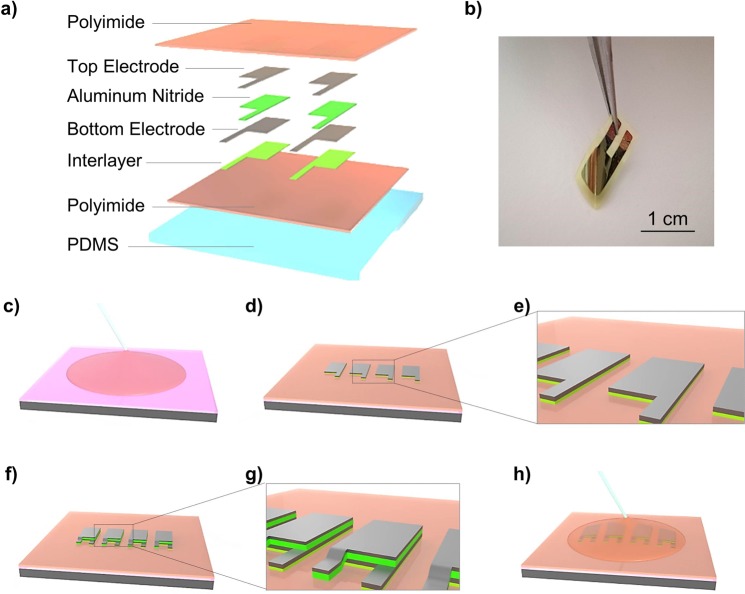
Flexible PSP-20. In the figure a schematic illustration (**a**) of the sensor heterostructure with the definition of the different layers is reported. (**b**) A freestanding PSP-20 image taken after the detachment from the rigid silicon substrate is shown. (**c**–**h**) The fabrication processing scheme for the AlN based smart patch is represented.

The piezoelectric sensor used for the vascular graft monitoring is based on a thin films heterostructure (Fig. 5a) and fabricated by standard micromachining process. Polyimide has been chosen as main structural layer for the growth of the entire sensor structure. Indeed, it has been already successfully exploited for AlN-based devices^29–32^ for its desirable structural and mechanical properties. In order to make the final device compliant and not intrusive with the graft walls, polyimidic acid based solution (instead of commercial Kapton foil) has been employed, allowing to strictly control the properties of the deposited film in terms of thickness and smoothness.

A 4 μm-thick layer of polyimide (PI2555®, HD Microsystem) has been used as a substrate for the multilayered structure consisting of aluminum nitride-interlayer (AlN-IL, 120 nm), molybdenum bottom electrode (Mo, 200 nm), piezoelectric aluminum nitride (AlN, 1 µm) and molybdenum top electrode (Mo, 200 nm) stack. The adhesion of the structure and texture orientation on the polymeric substrate has been guaranteed by the presence of the thin interlayer of AlN under the Mo bottom electrode^30^, promoting a well oriented growth and a high continuity through the single nanograin of the piezoelectric AlN.

The main steps of the micro-fabrication protocol of the sensor are illustrated in Fig. 5c–h. The sensor fabrication starts with the spinning of a thin film of poly(methyl methacrylate) (PMMA, 950PMMA® MicroChem, about 1 µm) on a silicon support; such film is used as sacrificial layer between Si and polyimide, allowing the final detachment of the freestanding devices at the end of the entire process. PI2555 is spun on PMMA and cured (at 130 °C for 60 minutes on hot plate and with a ramp up to 250 °C for 90 minutes in oven) in order to grow the first structural layer of polyimide. The stack of AlN-IL/Mo/AlN/Mo is deposited by reactive sputtering in two separate runs in order to simplify the patterning process. Both AlN layers are deposited at room temperature by using a high-purity Al target (99.9995%) with a 1:1 gas mixture ratio (N_2_:Ar) at a working pressure of 2.8 × 10^−3^ mbar, with a deposition rate of 17 nm/min, under a DC-pulsed power of 750 W and 1000 W for AlN-IL and piezoelectric AlN, respectively. Mo layers are sputtered using a pure Mo target (99.95%) in Ar atmosphere under DC power of 200 W and a working pressure of 5 × 10^−3^ mbar, with a deposition rate of 10 nm/min. AlN interlayer and bottom Mo electrode are sputtered directly on the polymeric substrate in the first single deposition run. The thin films AlN-IL/Mo has been patterned in rectangular geometries by dry etching, using inductively coupled plasma (ICP) etching system with defined gas mixtures of BCl_3_ and Ar, properly optimized to selectively etch these two materials. AlN piezoelectric thin film and Mo top electrode are then deposited by the second deposition run and patterned by ICP, exploiting the same BCl_3_ – Ar gas mixtures of the previous etching processes.

An insulating film of polyimide is then conformally deposited to mechanically protect the active area and make it waterproof, preventing short-circuits. At this stage, the device is detached from the rigid support by dipping the sample in an acetone bath for 10 minutes in order to melt the PMMA sacrificial layer. The freestanding structure (Fig. 5b) is finally transferred on an additional thin layer of PDMS (15 µm) to increases the flexibility of the overall system, allowing a perfect adhesion of the sensor to the vascular graft.

To guarantee a perfect adhesion between this additional flexible layer and the PI based transducer, both surfaces are chemically treated: the PDMS top surface is functionalized in a solution of adhesion promoter (VM651®, HD MicroSystem); in parallel, the device is dipped in potassium hydroxide (KOH) and hydrochloric acid (HCl) solutions to chemically activate the PI surface, and to promote the adhesion to the PDMS substrate. The total thickness of the final device, including the PDMS sheet, is about 20 µm, with an active area extension of 4 mm × 5 mm. Vias are opened, using O_2_ plasma, through the top polyimide coating, in order to guarantee electrical connections. The freestanding sensor, is then glued on the graft using a PVA-based adhesive layer.
